# Improved outcome of children with relapsed/refractory acute myeloid leukemia by addition of cladribine to re‐induction chemotherapy

**DOI:** 10.1002/cam4.3681

**Published:** 2021-01-24

**Authors:** Min Ruan, Li‐Peng Liu, Ao‐Li Zhang, Ben Quan Qi, Fang Liu, Tian‐Feng Liu, Xiao‐Ming Liu, Xiao‐Juan Chen, Wen‐Yu Yang, Ye Guo, Li Zhang, Yao Zou, Yu‐Mei Chen, Xiao‐Fan Zhu

**Affiliations:** ^1^ Division of Pediatric Blood Diseases Center State Key Laboratory of Experimental Hematology National Clinical Research Center for Blood Diseases Institute of Hematology & Blood Diseases Hospital Chinese Academy of Medical Sciences & Peking Union Medical College Tianjin China

**Keywords:** acute myeloid leukemia, children, cladribine, refractory, relapsed

## Abstract

**Background:**

The preferred salvage treatment for children with relapsed/refractory acute myeloid leukemia (R/R‐AML) remains unclear. The combination of cladribine/Ara‐C/granulocyte‐colony stimulating factor and mitoxantrone (CLAG‐M) shown promising results in adult R/R‐AML. We aim to investigate the efficacy and safety of CLAG‐M versus mitoxantrone/etoposide/cytarabine (MEC) or idarubicin/etoposide/cytarabine (IEC) in R/R‐AML children.

**Methods:**

Fifty‐five R/R‐AML children were analyzed. The overall response rate (ORR), overall survival (OS), and progression‐free survival (PFS) at 3‐year were documented. Karyotype or mutations status were summarized as different risk groups.

**Results:**

The ORR was achieved in 80% (16/20) and 51% (18/35) of patients after one‐cycle of CLAG‐M and MEC/IEC treatment (*p* < 0.001). The CLAG‐M group's OS (66.8% ± 16.2% vs. 40.4% ± 10.9%, *p* = 0.019) and PFS (52.6% ± 13.7% vs. 34.9% ± 9.1%, *p* = 0.036) at 3‐year was significantly higher than the MEC/IEC group. In high‐risk patients, 33.3% experienced progression of disease (PD) and 22.2% dead in CLAG‐M group, while 50% experienced PD and 43.8% dead in MEC/IEC. When it comes to low‐risk group, none of them in CLAG‐M experienced PD or death, while up to 50% of patients received MEC/IEC suffered PD, and all of them died eventually. Similar results were also found in the intermediate‐risk group. Surprisingly, the presence of *FLT3‐ITD* was associated with poor outcome in both groups. The most common adverse events were hematologic toxicities, and the incidence was similar in both group.

**Conclusions:**

CLAG‐M group demonstrated effective palliation along with acceptable toxicity in R/R‐AML patients. However, patients with *FLT3‐ITD* may benefit less from CLAG‐M, owing to higher PD rate and all‐cause mortality than other patients.

## Graphical abstract

CLAG‐M regimen is a more valid option with similar adverse events as a salvage treatment for R/R‐AML in children when compared with conventional chemotherapy no matter in which risk group.

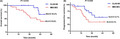

## 1 INTRODUCTION

Acute myeloid leukemia (AML), characterized by clonal expansion of abnormal differentiation of myeloid precursors, is a rare disease in children.^1^, ^2^ Despite the strides made in our understanding and treatment of AML in children in the past decades, concern about relapsed/refractory AML(R/R‐AML) in pediatric patients were still expressed.^3^, ^4^ Prognosis for this subgroup remains poor, with only 20%–30% event‐free survival (EFS).^5^, ^6^

Although the combination of cytarabine (Ara‐C) and anthracycline has been regarded as the standard of care for remission induction of AML patients, they did not improve outcomes in R/R‐AML patients. Cladribine represent a novel cytotoxic agent of salvage treatment with activity against R/R‐AML.^7^ Pharmacologic studies showed that prior administration of cladribine increases the intracellular uptake of cytarabine and the accumulation of cytarabine‐triphosphate (Ara‐CTP) in AML blasts both in vitro and ex vivo.^8^ Previous study also reported that the combination of cladribine, Ara‐C, granulocyte‐colony stimulating factor (G‐CSF), and mitoxantrone (CLAG‐M) has shown some promising results in adult R/R‐AML in comparison to conventional induction using mitoxantrone, etoposide, and Ara‐C.^9^, ^10^ Moreover, MEC (mitoxantrone, etoposide, and cytarabine) or IEC (idarubicin, etoposide, and cytarabine) is also sometimes used as a primary drug in a backbone of R/R‐AML patients with a CR rates of 18%–66% and 30‐day all‐cause mortality of 7%–11%.^11^, ^12^

Until now, few data are available in pediatric R/R‐AML regarding CLAG regimen. The condition in children R/R‐AML differ from those in adults, so this difference showed the difficulty of translating data derived from adults into the clinical practice of children management. Therefore, we aim to analyze the efficacy of CLAG‐M regimen in comparison to conventional chemotherapy in pediatric R/R‐AML patients.

## 2 MATERIALS AND METHODS

### 2.1 Study population

For this retrospective analysis, the source population included 70 children (age <18 years) with R/R‐AML at the two sites of the Division of Pediatric Blood Diseases Center in Institute of Hematology and Blood Diseases Hospital, Chinese Academy of Medical Sciences & Peking Union Medical College between Jan 2015 and Jan 2019. Of the 70 cases of R/R‐AML, salvage treatments of CLAG‐M or MEC/IEC had been abandoned before it started in 2 and 13 patients when they were first diagnosed as refractory AML or experienced relapse separately. So we had to exclude these 15 cases from the analysis. Out of 15 patients who discontinued medication before salvage treatment, 55 consecutive patients carrying the diagnosis of R/R AML who received MEC/IEC or CLAG‐M as salvage chemotherapy at any of the two sites of the Institute of Hematology and Blood Diseases Hospital, Chinese Academy of Medical Sciences & Peking Union Medical College, and the CLAG‐M or MEC/IEC protocol was used on basis of drug availability and not prespecified in this study. Moreover, both MEC/IEC and CLAG‐M regimens were available for eight patients at the same time, and these patients were randomly assigned to MEC/IEC or CLAG‐M group. The data collected included information regarding age, sex, peripheral blood (PB), white blood cell counts (WBC), blast percentages in bone marrow (BM), chromosome karyotypes, and gene mutation signatures.

The study design and methods complied with the Declaration of Helsinki and was approved by the Ethics Committee and Institutional Review Board of Institute of Hematology and Blood Diseases Hospital, Chinese Academy of Medical Sciences & Peking Union Medical College. Informed consent was obtained from all subjects.

### 2.2 Chemotherapy regimen

All patients in this study treated with CLAG‐M received cladribine 5 mg/m^2^ intravenously (IV) on days 1–5, Ara‐C 2 g/m^2^ IV on days 1–5, G‐CSF 300 μg/m^2^ subcutaneously starting the day prior to chemotherapy (day 0) and continuing through the days 5, and mitoxantrone 10 mg/m^2^ IV on days 1–3.^13^ Patients received conventional salvage chemotherapy (MEC or IEC) included etoposide IV over 2 h at 150 mg/m^2^ on days 1–5, mitoxantrone IV at 5 mg/m^2^ on days 6–10, and cytarabine 200 mg/m^2^ on days 6–12, or etoposide IV over 2 h at 200 mg/m^2^ on days 8–10, idarubicin IV at 8 mg/m^2^ on days 1–3, and cytarabine IV at 500 mg/m^2^ on days 1–3 and days 8–10. For almost 4 months, caused by a shortage of mitoxantrone in our center, IEC regimen was used as a substitute of MEC regimen in three patients, which was introduced in AML99 protocol.^14^ The rest 32 patients underwent MEC as the salvage therapy.

### 2.3 Next‐generation sequencing

The molecular abnormalities of all patients was analyzed at the timepoint of before and after CLAG‐M or MEC/IEC salvage treatment. Genomic DNA was extracted using the EZNA blood DNA Midi Kit (Omega Bio‐Tek). DNA samples were sequenced using the MiSeq platform (Illumina), which is approached by next‐generation sequencing (NGS). Libraries were prepared with a custom amplicon panel targeting 39 genes as previously reported, with a median depth of 2000× (Data S1).^15^

### 2.4 Data collection and definitions

Complete response (CR) was defined as the morphologic remission (<5% BM blasts) and recovery of PB counts (absolute neutrophil count >1000/mm^3^, platelet count >100,000/mm^3^) following induction. Partial response (PR) is defined by a decrease in BM blasts by at least 50% and recovery of PB counts detailed in CR. All other patients not falling into the above categories were considered treatment failures (no response, NR). Overall response rate (ORR) was defined as a CR or PR after one‐cycle of salvage treatment in all patients.

Progression of disease (PD) was defined by reappearance of myeloid blasts in either PB or BM (aspirate/biopsy) after achieving a remission or gaining evidence of leukemia cell infiltration in other organs. Refractory disease was defined by primary resistance to initial induction therapy based on the DA “3 + 7” and persistent AML with a blast count in the bone marrow >5% following second induction cycle. PFS (progression‐free survival) was defined as the interval between CLAG‐M or conventional salvage chemotherapy (MEC/IEC) and the first documentation of PD or death at 3‐year timepoint. OS (overall survival) was defined from day of salvage induction to death from any cause or last follow‐up.

Karyotype or molecular abnormalities were summarized as low risk [t(8;21), *CBFβ/MYH11*, and inv(16)], high risk (*FLT3‐ITD*, *KMT2A*, *FUS‐ERG*, *NRAS*, *KRAS*, and complex karyotype abnormalities), or intermediate risk (karyotype or molecular abnormalities not mentioned in the low or high‐risk groups).

### 2.5 Statistical analysis

Patients were subdivided by conventional therapy: patients who received MEC/IEC and patients who received CLAG‐M. Statistically significant differences between responders and nonresponders to salvage chemotherapy were tested using Pearson chi‐squared test for categorical variables and the Mann–Whitney *U*‐test for continuous variables. Both OS and PFS were estimated using the Kaplan–Meier method and survival estimates were compared using the log‐rank test. Two‐tailed *p* = 0.05 was set as the statistical significance level. All statistical analysis was performed using GraphPad Prism 8.02 software (GraphPad Software Inc.) and SPSS 22.0 (IBM Corporation).

## 3 RESULTS

### 3.1 Baseline characteristics

Of 70 children with R/R‐AML, except with 15 cases of interruption treatment, all of the rest of 55 patients were included for analysis. Among them, 20 cases of R/R‐AML were enrolled in CLAG‐M group and 35 cases were put into conventional chemotherapy (MEC/IEC) group, the median follow‐up was 28 months (range 4–52 months). Among the population with CLAG‐M regimens, 12 patients underwent chemotherapy‐only while 15 patients in conventional treatment group were without hematopoietic stem cell transplantation (HSCT). The clinical and cytogenetic features of the whole cohort are showed in Table 1, the median patient age was 8 years old (range 1–15) and 38 patients (69.1%) were male. Among all included patients, 38 (69.1%) received the salvage chemotherapy owing to refractoriness to the first‐line therapy (DA “3 + 7”), and the remaining 17 (30.9%) children were relapsed. All these 17 patients relapsed after treated with first‐line chemotherapy, and none of them relapsed after HSCT. When compared the baseline characteristics between two groups, insignificant differences were found in age, gender, FAB classification, and indication for salvage regimens (refractory or relapsed).

**TABLE 1:** Clinical characteristics of patients with relapsed/refractory‐AML

	CLAG‐M (N = 20)	MEC/IEC (N = 35)	*p* value
Male, no. (%)	15 (75.0)	23 (65.7)	0.473
Age (years), median (range)	7 (1–14)	8 (1–15)	0.886
≤3‐years‐old, no. (%)	6 (30.0)	9 (25.7)	0.731
Disease status			
Refractory, no. (%)	11 (55.0)	27 (77.1)	0.087
Relapsed, no. (%)	9 (45.0)	8 (22.9)	
FAB classification			
M2, no. (%)	5 (25.0)	9 (25.7)	0.835
M4, no. (%)	6 (30.0)	7 (20.0)	
M5, no. (%)	8 (40.0)	16 (45.7)	
M7, no. (%)	1 (5.0)	3 (8.6)	
Cytogenetics before salvage treatment			
Low risk, no. (%)	4 (20.0)	6 (17.1)	0.963
Intermediate risk, no. (%)	7 (35.0)	13 (37.1)	
High risk, no. (%)	9 (45.0)	16 (45.7)	
Karyotype, no. (%)			
Normal, no. (%)	9 (45.0)	18 (51.4)	0.813
inv (16), no. (%)	3 (15.0)	6 (17.1)	
Complex karyotype, no. (%)	8 (40.0)	11 (31.4)	
Cytogenetic, mutation group, no. (%)			
*KMT2A*	1 (5.0)	3 (8.6)	0.624
*FLT3‐ITD*	4 (20.0)	6 (17.1)	0.792
*NRAS*/*KRAS*	2 (10.0)	3 (8.6)	0.859
*RUNX1*‐*RUNX1 T1* or *CBFβ*‐*MYH11*	4 (20.0)	6 (17.1)	0.792

Regarding the cytogenetic characterization, the distribution of karyotype and mutation status were similar in both groups (all *p* > 0.05). In particular, the mutation of FLT3 internal tandem duplication (ITD) was presented in 18.2% (10/55) of all patients while *NRAS*/*KRAS* and *KMT2A‐rearrangement* were found in 9.1% (5/55) and 7.3% (4/55) of included subjects. Core‐binding factor (CBF) alterations, namely *RUNX1‐RUNX1T1* or *CBFβ/MYH11* fusions, were found in 18.2% (10/55) children in whole cohort. According to cytogenetic status before salvage treatment, 4, 7, 9 patients underwent CLAG‐M and 6, 13, 16 patients underwent MEC/IEC were classified in low‐risk, intermediate‐risk, and high‐risk group, respectively. There existed insignificant difference between the CLAG‐M and MEC/IEC group in terms of risk classification (*p* = 0.963) (Table 1).

### 3.2 Therapeutic effect and prognostic analysis of two regimens

**FIGURE 1:**
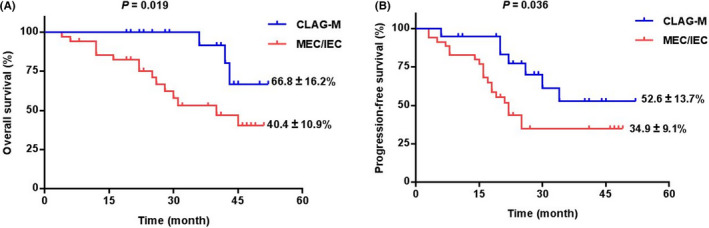
A, Overall survival (OS) analysis for the R/R‐AML children in two regimens. B, Progression‐free survival (PFS) analysis for the R/R‐AML children in two regimens

**FIGURE 2:**
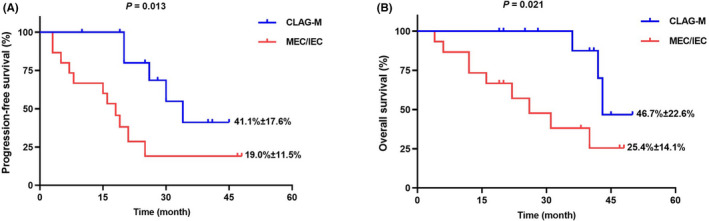
A, OS analysis for the R/R‐AML children without hematopoietic stem cell transplantation (HSCT) in two regimens. B, PFS analysis for the R/R‐AML children without HSCT in two regimens

Figure 1A illustrated that the CLAG‐M group's OS at 3‐year was significantly higher than the MEC/IEC group (66.8% ± 16.2% vs. 40.4% ± 10.9%, *p* = 0.019). Moreover, there was also significantly higher PFS at 3‐year in patients underwent CLAG‐M (52.6% ± 13.7% vs. 34.9% ± 9.1%, *p* = 0.036) (Figure 1B). What is more, other than three patients with IEC, we compared 32 patients underwent MEC with 20 patients in CLAG‐M group, lower OS and PFS was documented in MEC group (*p* = 0.023 and *p* = 0.050) (Figure S1a,b). According to Figure 2A,B, in 27 patients who did not receive HSCT, there was also significantly poorer OS and PFS in MEC/IEC group when compared with CLAG‐M group (both *p* < 0.05), which was similar to those obtained in all patients.

The CR rate was achieved in 75% (15/20) of patients after one‐cycle of CLAG‐M and remained in remission during follow‐up, whereas one patient achieved PR after treatment, so the ORR of CLAG‐M in R/R‐AML children is 80% (16/20). As for 35 patients underwent MEC/IEC, only 23% (8/35) patients achieved CR and 28% (10/35) patients had PR after one‐cycle of MEC/IEC treatment, which resulting in an ORR of 51% (18/35). Thus, the ORR of R/R‐AML children received CLAG‐M regimens was significantly higher than MEC/IEC group (*p* < 0.001) (Table 2). In 17 of 55 patients experienced relapse after first‐line therapy, and the ORR of relapsed patients underwent CLAG‐M was 77.8% (7/9) versus 50% (4/8) in MEC/IEC group (*p* = 0.232). Although there existed insignificant differences between two groups, the ORR of CLAG‐M was higher than conventional regimens.

**TABLE 2:** Individual patient data of the clinical outcome after salvage treatment

	Patient number	Risk group	Number of Mutation	Driver mutation	Allele frequency before treatment	Allele frequency after treatment	Response after treatment	Progression of disease	Alive during follow‐up
CLAG‐M	1	High risk	2	*NRAS p. G13D*	17.0%	0%	CR	Yes	Alive
CLAG‐M	2	High risk	3	*WT1*	52.1%	0%	CR	No	Alive
CLAG‐M	3	High risk	3	*FLT3‐ITD*	23.0%	0%	CR	No	Alive
CLAG‐M	4	High risk	2	*FLT3‐ITD*	10.0%	0%	CR	No	Alive
CLAG‐M	5	Low risk	1	*RUNX1‐RUNX1 T1*	138.1%	0%	CR	No	Alive
CLAG‐M	6	High risk	3	*NRAS p. Q61 K*	13.0%	0%	CR	No	Alive
CLAG‐M	7	High risk	1	*FLT3‐ITD*	31.0%	37.5%	NR	Yes	Dead
CLAG‐M	8	Intermediate risk	1	*WT1*	78.3%	84.3%	NR	Yes	Dead
CLAG‐M	9	Intermediate risk	2	*KRAS p. G12 V*	4.8%	0%	CR	No	Alive
CLAG‐M	10	Intermediate risk	2	*CEBPA p.Q312*	50.0%	0%	CR	No	Alive
CLAG‐M	11	Low risk	1	*CBFB‐MYH11*	204.6%	0%	CR	No	Alive
CLAG‐M	12	Intermediate risk	1	*NPM1*	15.8%	0%	CR	No	Alive
CLAG‐M	13	Low risk	3	*RUNX1‐RUNX1 T1, KIT*	65.6%	41.2%	NR	No	Alive
CLAG‐M	14	High risk	2	*FLT3‐ITD*	0.88%	2.7%	NR	Yes	Dead
CLAG‐M	15	Low risk	2	*CBFB‐MYH11*	66.8%	0%	CR	No	Alive
CLAG‐M	16	Intermediate risk	1	*IDH2 p.R140Q*	38.4%	0%	CR	Yes	Alive
CLAG‐M	17	Intermediate risk	3	*C‐KIT p.A755 T*	50.6%	0%	CR	Yes	Alive
CLAG‐M	18	High risk	2	*P53*	20.8%	0%	CR	No	Alive
CLAG‐M	19	High risk	2	*KMT2A‐AF9*	14.1%	0%	CR	No	Alive
CLAG‐M	20	Intermediate risk	1	*IDH1 p.R132C*	4.3%	0.4%	PR	No	Alive
MEC	1	Intermediate risk	2	*NRAS p. G13D*	1.7%	1.0%	NR	Yes	Alive
MEC	2	High risk	2	*FLT3‐ITD*	0.2%	0%	CR	No	Alive
MEC	3	Intermediate risk	2	*KRAS p. G13D*	12.8%	2.2%	PR	Yes	Alive
MEC	4	Intermediate risk	2	*WT1*	89.2%	64.8%	NR	Yes	Dead
MEC	5	Intermediate risk	1	*WT1*	61.0%	2.31%	PR	No	Alive
IEC	6	High risk	0	*—*	*—*	*—*	CR	No	Alive
MEC	7	Low risk	1	*RUNX1‐RUNX1 T1*	45.2%	6.4%	PR	Yes	Alive
MEC	8	Intermediate risk	2	*FLT3‐TKD*	35.0%	0%	CR	No	Alive
MEC	9	High risk	1	*NRAS p. G13 V*	46.1%	41.2%	NR	No	Alive
MEC	10	Low risk	1	*RUNX1‐RUNX1 T1*	53.0%	11.0%	PR	No	Alive
MEC	11	Low risk	2	*CBFB‐MYH11*	272.5%	8.1%	PR	No	Alive
MEC	12	Intermediate risk	1	*RUNX1 p.Q397R*	55.5%	0%	CR	No	Alive
MEC	13	Intermediate risk	1	*CEBPA p.Q312*	15.0%	0%	CR	No	Alive
IEC	14	High risk	1	*FLT3‐TKD*	2.1%	0.9%	PR	Yes	Alive
MEC	15	Intermediate risk	0	*—*	*—*	*—*	NR	Yes	Dead
MEC	16	Intermediate risk	0	*—*	*—*	*—*	NR	Yes	Dead
MEC	17	Intermediate risk	0	*—*	*—*	*—*	CR	Yes	Dead
MEC	18	High risk	1	*KMT2A‐AF4*	31.1%	48.1%	NR	Yes	Dead
MEC	19	High risk	1	*KMT2A‐AF9*	22.3%	19.2%	NR	Yes	Dead
MEC	20	High risk	1	*KMT2A‐AF9*	41.1%	37.7%	NR	Yes	Dead
MEC	21	High risk	0	*—*	*—*	*—*	CR	No	Alive
MEC	22	High risk	1	*RUNX1‐RUNX1 T1*	34.8%	37.8%	NR	No	Alive
MEC	23	Intermediate risk	0	*—*	*—*	*—*	NR	Yes	Alive
MEC	24	Intermediate risk	0	*—*	*—*	*—*	PR	No	Alive
MEC	25	High risk	1	*FLT3‐ITD*	46.2%	37.8%	NR	Yes	Dead
IEC	26	High risk	1	*FLT3‐ITD*	57.8%	41.9%	NR	Yes	Dead
MEC	27	High risk	2	*FLT3‐ITD*	48.1%	2.3%	PR	No	Alive
MEC	28	High risk	2	*FLT3‐ITD*	22.5%	19.7%	NR	Yes	Dead
MEC	29	High risk	2	*KRAS p. G12 V*	62.3%	3.7%	PR	No	Alive
MEC	30	Intermediate risk	0	*‐*	‐	‐	NR	Yes	Dead
MEC	31	Low risk	1	*RUNX1‐RUNX1 T1*	162.9%	87.1%	NR	Yes	Dead
MEC	32	Low risk	2	*RUNX1‐RUNX1 T1, KIT*	119.4%	77.2%	NR	Yes	Dead
MEC	33	Low risk	2	*RUNX1‐RUNX1 T1, KIT*	67.8%	37.5%	NR	Yes	Dead
MEC	34	High risk	1	*FUS‐ERG*	21.2%	0%	CR	No	Alive
MEC	35	High risk	1	*FLT3‐ITD*	31.9%	5.2%	PR	Yes	Dead

Abreviation: CR: complete response; NR: No response; PR: partial response.

Table 2 also revealed that all 20 patients in CLAG‐M and 27 patients in MEC/IEC group had mutations before salvage treatment, for the eight remaining patients in MEC/IEC group, no driver mutation was initially detected at that timepoint. Comprehensive cytogenetic analysis revealed that in all nine patients received CLAG‐M in high‐risk group, 33.3% (3/9) patients experienced PD and 22.2% (2/9) dead, while among 16 high‐risk patients received MEC/IEC, 50% (8/16) of suffered PD and 43.8% (7/16) dead. However, four patients underwent CLAG‐M and six underwent MEC/IEC harbored *FLT3*‐*ITD* alterations, the presence of this mutation was associated with lower ORR (50% and 50%), higher PD rate (50% and 66.7%), and higher all‐cause mortality rate (50% and 66.7%) in both CLAG‐M and MEC/IEC group.

When it comes to low‐risk patients with CBF‐AML (*RUNX1‐RUNX1T1* or *CBFβ/MYH11*), none (0/4) of them in CLAG‐M experienced PD or death, while up to 50% (3/6) of patients received MEC/IEC suffered PD within 6 month, and all of them died eventually. Similar results were also found in the intermediate‐risk group, although 42.9% (3/7) patients in CLAG‐M group had PD, only 14.3% (1/7) children died during follow‐up. Surprisingly, 61.5% (8/13) patients experienced PD and 38.5% (5/13) died in salvage MEC/IEC group.

We next compared responders (CR+PR) and nonresponders (NR) in both group to assess the potential factors influencing response of treatment. However, there was no difference in the number of mutations between them. What is more, there existed insignificant differences between the responders and nonresponders regarding baseline clinical characteristics including age, white blood cell count, and BM blasts before treatment (all *p* > 0.05). However, by evaluating the molecular responses, we were able to identify patients who experienced molecular remissions and went on to have a better OS and PFS no matter in CLAG‐M or MEC/IEC group.

### 3.3 Adverse events

All the adverse response was reported regardless of attribution and include events that occurred during treatment and follow‐up (Table 3). The most common grade 3–4 adverse events were hematologic toxicities (such as anemia, neutropenia, and thrombocytopenia), all 55 patients in both groups experienced these adverse events. Febrile neutropenia was more frequent in the MEC/IEC group than in the CLAG‐M group although without statistical significance (*p* = 0.386), and the frequency of bleeding and infection was low and similar between groups (*p* = 0.859 and *p* = 0.386). Table 3 also indicated that the durations of Grade 3–4 hematologic toxicities was also comparable between two groups in this study (*p* = 0.811). All adverse events were controllable in both groups, and recovered after symptomatic treatment.

**TABLE 3:** Summary of grade 3/4 adverse events

Adverse events	CLAG‐M (n = 20)	MEC/IEC (n = 35)	*p* value
Hematologic toxicities			
Hemoglobin<60 g/L, no. (%)	20 (100)	35 (100)	>0.999
Neutrophil count<0.5 × 10^9^/L, no. (%)	20 (100)	35 (100)	>0.999
Platelet count<20 × 10^9^/L, no. (%)	20 (100)	35 (100)	>0.999
Duration of grade 3/4 hematological toxicities	17 (8–33)	16 (5–30)	0.811
Non‐hematologic toxicities			
Bleeding, no. (%)	2 (10.0)	3 (8.6)	0.859
Infection, no. (%)	9 (45.0)	20 (57.1)	0.386
Febrile neutropenia, no. (%)	11 (55.0)	15 (42.9)	0.386
Classification of infectious diseases			
Bacteria	7 (35.0)	16 (45.7)	0.438
Fungi	2 (10.0)	4 (11.4)	0.870
Virus	0 (0)	0 (0)	>0.999

## 4 DISCUSSION

Because of the inferior outcome of R/R‐AML children when treated with standard chemotherapy of DA 3 + 7 induction, this cohort prompts the application of investigational treatment. In this study, we identified that approximately 75% of R/R‐AML children who received one‐cycle CLAG‐M salvage therapy achieved a complete remission when compared with only 23% in MEC/IEC chemotherapy, moreover, a better prognosis was also obtained in CLAG‐M group with separate risk group. However, patients with *FLT3‐ITD* may benefit less from CLAG‐M, which was similar to MEC/IEC regimens, owing to higher PD rate and all‐cause mortality than other patients.

Various salvage treatments are emerging for R/R‐AML children. However, optimal re‐induction therapy is still unknown. Among current therapy protocols, hematopoietic stem cell transplantation (HSCT) remains the standard procedure, increasing short‐ and long‐term prognosis of these patients.^16^ Therefore, the use of CLAG‐M or MEC/IEC might be of particular importance when HSCT for various reasons cannot be done, such as lack of an HLA‐matched donor that cannot be foreseen when the confirmation of refractory or relapse comes. The important feature of cladribine is likely to influence the cellular uptake action of cytarabine and accumulation of Ara‐CTP in circulating blasts by more than 50%, which ensured a more complete therapeutic effect of CLAG‐M regimen.^17^, ^18^ Since then, CLAG regimens have been investigated mostly in adult patients with AML, a recent study of CLAG‐M chemotherapy in adults with AML reported CR rates of 58% with 30‐day treatment‐related mortality of 7%.^10^

Since then, only one study with small sample size of 12 children have analyzed the efficacy of CLAG regimen in R/R‐AML children.^19^ They reported that the 3‐year PFS and OS of children received CLAG were 44.4 ± 15.7% and 59.5 ± 16.2%, which were lower than our result. In a study conducted by Wierzbowska et al., the addition of mitoxantrone to create CLAG‐M regimens was reported to increase the CR rate by 10%–15% when compared with CLAG only in adults with R/R‐AML.^20^ As if to prove the point, our findings may indicated the better efficacy of CLAG‐M than previously existing treatments like CLAG treatment. Moreover, this research did not include molecular results, and cytogenetic characterization may be recognized as an independent marker conferring vastly different prognosis in patients with R/R‐AML. In view of this, a research focusing on these issues may bring some illumination for better treatment in various kinds of R/R‐AML children.

Although a significant higher number of patients in CLAG‐M group achieved CR and had a better prognosis after one‐cycle chemotherapy than conventional chemotherapy, we showed that CLAG‐M regimen did not yield improvements for every R/R‐AML patients, and the response rate and prognosis remains disappointing in *FLT3‐ITD* patients no matter in the CLAG‐M group or MEC/IEC group. *FLT3‐ITD* is a common mutation and with poor prognosis in children with AML, and patients with *FLT3‐ITD* exhibited limited progress in prognosis notwithstanding several kinds of chemotherapy according to recent studies.^21^, ^22^ In view of this, *FLT3‐ITD* mutation's presence was associated with poorer prognosis, and intensive chemotherapy combined with tyrosine kinase inhibitors (TKI), such as midostaurin, quizartinib, or sorafenib may be required followed by HSCT.

There is also a limitation in our study. Although our results were with statistical significance, the small sample size in the study suggests that further study with larger sample size is needed to confirm this results. Nowadays, there is an ongoing prospective, randomized and multicenter study right now to investigate the potential efficacy of CLAG‐M in pediatric R/R‐AML.

In conclusion, on basis of significant differences in outcomes among patients who received CLAG‐M versus MEC/IEC, we believe that the CLAG‐M regimen may have a potential of being regarded as an effective treatment along with acceptable toxicity as a salvage therapy for R/R‐AML in children when compared with conventional chemotherapy no matter in which risk group. Despite some progress were made, outcomes in *FLT3*‐*ITD* carriers remain unsatisfactory, and some effective treatment is still warranted.

## CONFLICT OF INTEREST

None.

## AUTHOR CONTRIBUTIONS

Conception and design: M.R, L.P.L, X.F.Z. Development of methodology: X.J.C, W.Y.Y, X.M.L, Y.G. Acquisition of data: A.L.Z, B.Q.Q, F.L, T.F.L, L.Z, Y.Z. Writing, review, and/or revision of the manuscript: M.R, X.F.Z, Y.M.C

## Supporting information

Fig S1

Supplementary Material

## Data Availability

The data that support the findings of this study are available from the corresponding author upon reasonable request.
