# Supplementary figures and images for: Improved outcome of children with relapsed/refractory acute myeloid leukemia by addition of cladribine to re‐induction chemotherapy
Source: Cancer Med. 2021 Jan 24;10(3):956–64. doi: 10.1002/cam4.3681 (PMC7897947; doi:10.1002/cam4.3681)

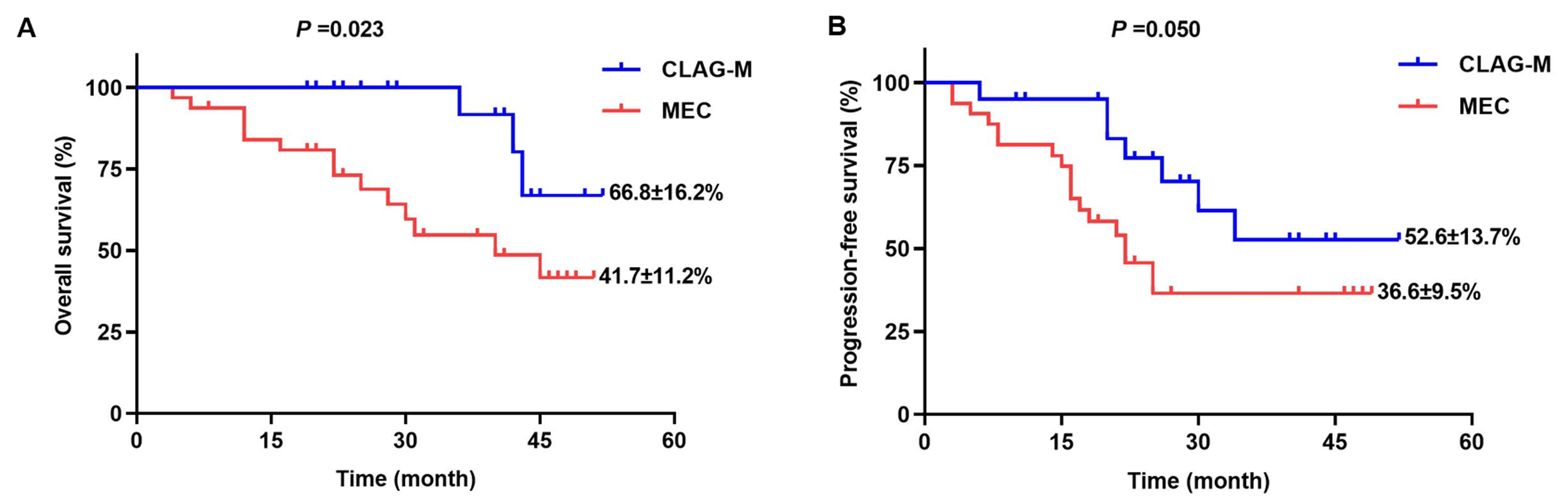

Supplement: Supplementary file 1 — Fig S1 [file CAM4-10-956-s001.tif]
