# Supplementary material for: Improved outcome of children with relapsed/refractory acute myeloid leukemia by addition of cladribine to re‐induction chemotherapy
Source: Cancer Med. 2021 Jan 24;10(3):956–64. doi: 10.1002/cam4.3681 (PMC7897947; doi:10.1002/cam4.3681)
Supplement: Supplementary file 2 — Supplementary Material [file CAM4-10-956-s002.docx]

A list of 39 genes evaluated by NGS: The genetic mutation status of all patients was analyzed at the time of admission. Genomic DNA was extracted using the EZNA blood DNA Midi Kit (Omega Bio-Tek, Norcross, GA, USA). Whenever possible, BM samples were used for analyses. DNA samples were sequenced using the MiSeq platform (Illumina, San Diego, CA), which is a custom, targeted, amplicon-based sequencing approach by NGS. Libraries were prepared with a custom amplicon panel targeting AML1/ETO (RUNX1/ RUNX1T1), CBFβ-MYH11, DNMT3A, ETNK1, ETV6, EZH2, FLT3, GATA2, IDH1, IDH2, IL7R, JAK2, ASXL1, ASXL2, BCOR, BCORL1, BIRC3, BRAF, CALR, CBL, CDKN2A, KIT, KMT2A (MLL), KRAS, MPL, MYD88, NOTCH1, NRAS, PAX5, PDGFRA, PDGFRB, PTEN, PTPN11, SETBP1, SETD2, STAG2, TET2, TP53 and WT1, with a median depth of 2000×.

Table S1. Clinical demographics between responders and non-responders of salvage chemotherapy.

|  | CLAG-M  (N=20) | |  | MEC/IEC  (N=35) | |  |
| --- | --- | --- | --- | --- | --- | --- |
|  | Responders  (N=16) | Non-responders  (N=4) | *P* value | Responders  (N=18) | Non-responders  (N=17) | *P* value |
| Male, no. (%) | 11(68.8) | 4 (100.0) | 0.197 | 13 (72.2) | 10 (58.8) | 0.404 |
| Age (years), median (range) | 6 (1-14) | 9 (4-10) | 0.694 | 8 (1-15) | 9 (3-14) | 0.333 |
| White blood cell count, ×10^9^/L | 3.6 (0.9-107.2) | 7.3 (1.8-54.4) | 0.682 | 4.3 (0.8-58.8) | 3.4 (0.4-33.1) | 0.702 |
| BM blast, median (range) | 20 (6-85) | 49 (22-87) | 0.140 | 29 (6-98) | 40 (6-95) | 0.198 |
| Number of mutation | 2 (1-3) | 1 (1-2) | 0.083 | 1 (0-2) | 1 (0-2) | 0.548 |
